# Palliative psychiatry for severe persistent mental illness as a new approach to psychiatry? Definition, scope, benefits, and risks

**DOI:** 10.1186/s12888-016-0970-y

**Published:** 2016-07-22

**Authors:** Manuel Trachsel, Scott A. Irwin, Nikola Biller-Andorno, Paul Hoff, Florian Riese

**Affiliations:** Institute of Biomedical Ethics and History of Medicine, University of Zurich, Winterthurerstrasse 30, CH-8006 Zurich, Switzerland; Supportive Care Services, Samuel Oschin Comprehensive Cancer Institute, Cedars-Sinai Health System, Los Angeles, CA USA; Department of Psychiatry, Cedars-Sinai Health System, Los Angeles, CA USA; Psychiatric University Hospital Zurich, Zurich, Switzerland; URPP “Dynamics of Healthy Aging”, University of Zurich, Zurich, Switzerland

**Keywords:** Palliative care, End of life, Quality of life, Futility, Severe persistent mental illness, Terminal care, Psychiatry, Palliative sedation

## Abstract

**Background:**

As a significant proportion of patients receiving palliative care suffer from states of anxiety, depression, delirium, or other mental symptoms, psychiatry and palliative care already collaborate closely in the palliative care of medical conditions. Despite this well-established involvement of psychiatrists in palliative care, psychiatry does not currently explicitly provide palliative care for patients with mental illness outside the context of terminal medical illness.

**Discussion:**

Based on the WHO definition of palliative care, a, a working definition of palliative psychiatry is proposed. Palliative psychiatry focuses on mental health rather than medical/physical issues. We propose that the beneficiaries of palliative psychiatry are patients with severe persistent mental illness, who are at risk of therapeutic neglect and/or overly aggressive care within current paradigms. These include long-term residential care patients with severe chronic schizophrenia and insufficient quality of life, those with therapy-refractory depressions and repeated suicide attempts, and those with severe long-standing therapy-refractory anorexia nervosa. An explicitly palliative approach within psychiatry has the potential to improve quality of care, person-centredness, outcomes, and autonomy for patients with severe persistent mental illness.

**Conclusions:**

The first step towards a palliative psychiatry is to acknowledge those palliative approaches that already exist implicitly in psychiatry. Basic skills for a palliative psychiatry include communication of diagnosis and prognosis, symptom assessment and management, support for advance (mental health) care planning, assessment of caregiver needs, and referral to specialized services. Some of these may already be considered core skills of psychiatrists, but for a truly palliative approach they should be exercised guided by an awareness of the limited functional prognosis and lifespan of patients with severe persistent mental illness.

## Background: psychiatry in palliative care versus palliative care in psychiatry

Psychiatry and palliative care share common ground: both disciplines have evolved historically from internal medicine, are grounded in the biopsychosocial model and usually operate within multiprofessional teams [1]. Already, psychiatrists collaborate closely in the palliative care of medical conditions, as a significant proportion of patients receiving palliative care suffer from states of anxiety (approx. 30 %) [2], depression (approx. 38 %) [3], delirium (between 20–45 %) [4], and other mental symptoms approaching the threshold of mental disorder [5, 6]. Overall, collaboration between the fields of psychiatry and palliative care has grown significantly in most developed countries over the last two decades [7] and is frequently practised under the rubric of palliative care psychiatry [8] or psycho-oncology [9]. Indeed, provision of psychiatric, psychotherapeutic, or psychosocial care is considered an indicator for high-quality palliative care in cases of advanced medical disease [10]. Beyond the context of medical illness, however, current psychiatry practice does not explicitly provide palliative care or name it as such. However, on reflection, several clinical approaches in contemporary psychiatry can already be considered palliative, as they aim at reducing symptoms and suffering from mental illness rather than seeking to achieve disease remission or disease modification, for example the recovery approach (for the relation between PP and recovery see “Risks of a palliative approach to psychiatry” below).

The Swiss Academy of Medical Sciences guidelines on palliative care specify several groups of psychiatric patients who can potentially benefit from such approaches: “Many psychiatric disorders can have a chronic course or are characterized by frequent relapses. In such cases a palliative approach is all the more important that does not primarily aim at fighting the disease but at optimal management of the symptoms and disability. Quality of life can often be improved and suicide risk can be reduced when palliative support and attention take place in addition to curative or disorder-specific treatments. Difficult situations arise in particular from: therapy-refractory depressions with repeated suicide attempts with intent to die; severe cases of schizophrenia with, from the patient’s perspective, insufficient quality of life; [and] severe anorexia” [translated by the authors] [11]. While there is no international consensus on a definition, the patient population referred to in these guidelines can be considered to be suffering from “severe persistent mental illness” (SPMI) [12, 13].

As well as provision of psychiatric palliative care for patients with SPMI, the Swiss Academy of Medical Sciences guidelines also acknowledge a need for improved somatic medical care for the mentally ill: “Mentally ill patients can also be affected by physical illness. There is a risk that their symptoms get overlooked or are not properly classified. In these situations, a close cooperation between psychiatrists/psychotherapists with specialists from other medical disciplines is necessary” [11]. Improved palliative care for physical illness in mental health patients is beyond the scope of this article but is undoubtedly of central importance, given the multiple.

comorbidities and higher-than-average mortality rates of patients with SPMI [14, 15]. In this article, we choose to focus on the palliative approach to mental health issues in patients with SPMI because, from our point of view, psychiatry itself corresponds in many respects to palliative care as psychiatric treatments are frequently non-curative.

## The scope of palliative care in psychiatry

**Fig. 1:**
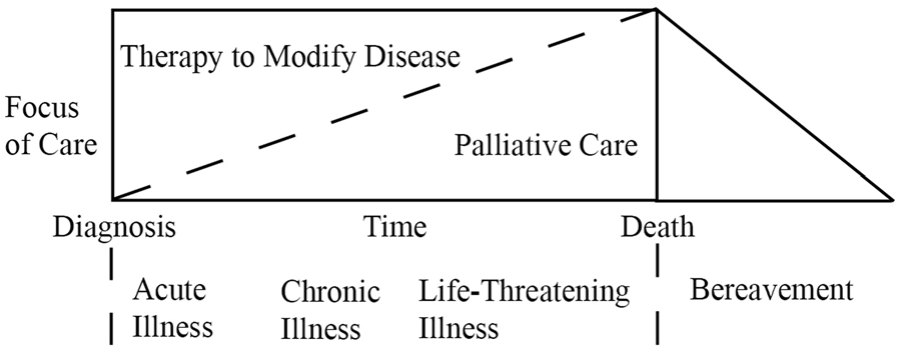
Palliative care model (adapted from Ferris et al., 2002) [16]

The range of palliative care approaches in medicine is very broad, from short-term, targeted measures for alleviation of distressing symptoms to palliative sedation as the ultima ratio. The common “palliative” denominator of such interventions is to accept that they help to stabilize or improve quality of life without necessarily modifying disease progression in the long term: some palliative interventions may even be undertaken at the conscious expense of potentially shortening remaining life expectancy. In practice, disease-modifying and palliative approaches are often carried out in parallel (see Fig. 1) [16]. According to such a definition, many, (if not nearly all) established interventions in psychiatry aiming to promote quality of life rather than remission can be considered palliative—for example, psychiatric long-term residential care for patients with clozapine resistant schizophrenia; [17] severe enduring anorexia nervosa where the decision is made to forego repeat hospitalizations with further cycles of involuntary refeeding; [18] and palliative sedation for therapy-refractory hopelessness or anxiety in the process of dying [19]. However, this way of thinking is rarely (if ever) explicitly acknowledged within the discipline of psychiatry.

## Definition and features of palliative psychiatry

Based on the World Health Organization [20] definition of palliative care, we propose a working definition of palliative psychiatry (PP) [1] as a starting point for discussing of the usefulness of palliative psychiatry as a new conceptual and clinical approach (Table 1).

**Table 1:** Working definition, features and examples of palliative psychiatry

Definition	
Palliative psychiatry (PP) is an approach that improves the quality of life of patients and their families in facing the problems associated with life-threatening severe persistent mental illness (SPMI) through the prevention and relief of suffering by means of a timely assessment and treatment of associated physical, mental, social, and spiritual needs. PP focuses on harm reduction and on avoidance of burdensome psychiatric interventions with questionable impact.	
Features of palliative psychiatry	
- Provides support in coping with and accepting of distressing mental symptoms	
- Affirms life but acknowledges that SPMI can be incurable	
- Intends neither to hasten nor to postpone death	
- Integrates the physical, psychological, social, and spiritual aspects of patient care	
- Offers a support system to help patients to live as actively as possible until death	
- Offers a support system to help family members to cope during patients’ SPMI	
- Uses a team approach to address the needs of patients and their families	
- Will enhance quality of life and may also positively influence the course of the SPMI	
- Is applicable in conjunction with other therapies oriented towards prevention, curation, rehabilitation, or recovery	

It has been argued that “in many aspects, psychiatry itself is a form of palliative care because psychiatric treatments are frequently not curative” [21]. However, we do not find it helpful to use the term *palliative psychiatry* for all kinds of psychiatric intervention, such as short-term “palliative” measures with limited risks (e.g. one-time dispensation of a benzodiazepine for acute anxiety). As a consequence, we propose to restrict the use of the term to SPMI with multiple comorbidities and higher-than-average mortality rates (e.g. schizophrenia [22], or anorexia nervosa [23]). Furthermore, we do not intend that palliative psychiatry should compete with or compromise other important conceptual advances in psychiatry, such as the recovery model (see “Risks of a palliative approach to psychiatry” below), but rather to complement them by acknowledging their possible futility in some cases. However, we would not call persons with SPMI “terminally ill”, as palliative care can be provided independent of concrete end-of-life situations. A palliative approach comes into play when a disease cannot (or can no longer) be treated with merely curative intent. Accordingly, the WHO definition of palliative care and the definition of palliative psychiatry proposed above do not require patients to be “terminally ill” to be eligible for palliative approaches that may be applied in conjunction with other therapies oriented towards prevention, curation, rehabilitation, or recovery.

Basic PP skills include ongoing communication of psychiatric diagnosis and prognosis, symptom assessment and management, support for advance (mental health) care planning, assessment of caregiver needs, and referral to specialized services [24]. Some of these may be considered core skills of a psychiatrist in any event, but in PP they need to be exercised “sub specie mortis” (that is, guided by an awareness of limited functional prognosis and lifespan).

While some forms of palliative treatment for mental illness are already well established, others are highly controversial (see Table 1). Whether palliative sedation—that is, “the use of sedative medications to relieve intolerable and refractory distress by the reduction in patient consciousness” [20] (p. 447)—should be justified for patients with SPMI in states of therapy-refractory hopelessness or anxiety is an ongoing discussion [25–28]. Furthermore, discussion of a palliative approach to SPMI is intricately linked to the issue of a “right to die” for the mentally ill. In The Netherlands and Belgium, respectively, physician-assisted suicide is allowed in non-terminal cases of “lasting and unbearable suffering” [29] or when the suffering is “constant” and “cannot be alleviated” [30]. According to a decision of the Swiss Federal Supreme Court, assisted suicide is also legal for patients with mental illness in Switzerland [31]. The ethical issues, as well as arguments for and against the extension of assisted-suicide rights to patients with SPMI, have been discussed elsewhere [32]. Notably, there is initial evidence that better palliative care strategies and services for persons with SPMI may lead to fewer requests for assisted suicide [33, 34].

## Benefits of a palliative approach to psychiatry

Although PP may not be appropriate for all patients with mental illness, it is our expectation that a substantial number of patients with SPMI may benefit from the approach. For example, between a fifth and a third of patients with schizophrenia (corresponding to approximately four to seven million people worldwide [35]) suffer from severe treatment-resistant schizophrenia. These patients may exhibit a high level of negative symptoms, leading to impaired quality of life and social functioning. In some of these cases, patient needs may not be met by current psychiatric services, and patients may be at risk of being abandoned or may face therapeutic neglect. Depending on the stage of illness, as an alternative to countless cycles of treatments that are ever less evidence-based but ever more burdensome and costly, psychiatrists could increasingly focus on promoting quality of life. By so doing, the therapeutic relationship may improve, with fewer dropouts from therapy as patients come to see the suggested palliative treatments as a better fit to their situation.

The hypothesis that a substantial number of patients with SPMI may benefit from PP remains to be empirically tested. Adopting a PP paradigm may further allow clinical trial designs to be tailored to SMI, potentially increasing opportunities for research participation and, in the longer run, increasing treatment options for this population. Additionally, establishing an explicitly palliative approach to psychiatry, with its own clinical techniques, concepts, and research opportunities, may also help to attract more psychiatrists and other mental health professionals to the treatment of SPMI, with indirect benefits for this particularly vulnerable patient population [1].

## Risks of a palliative approach to psychiatry

One possible reason for the current lack of explicitly palliative care directed at mental illness (especially SPMI) is the lack of consensus about the meaning of “futility” in this context. Despite the substantial body of literature on futility in somatic medicine [36], the concept has so far been discussed in relation to the treatment of mental illness only within the contexts of severe persistent anorexia nervosa and dementia [18, 37]. Lopez, Yager, and Feinstein [38], the first authors to link the terms “medical futility” and “psychiatry” in the title of an article, suggested the following criteria for treatment futility: (1) poor prognosis; (2) unresponsiveness to competent treatment; (3) continuing physiological and psychological decline; and (4) the appearance of an inexorable and terminal course. In our view, the discussion around futility in psychiatry can be substantially advanced by the development of evidence-based disease staging for mental illness, similar to those in cancer care [39]. For example, while duration of illness, previous treatment attempts, or level of associated disability must be taken into account, it “is clear that a 14 year old adolescent with a 3 month history of anorexia nervosa would present differently to a 40 year old woman who has battled the illness for 25 years with multiple hospital admissions and has attempted cognitive behaviour therapy several times” [40] (p. 1). At present, for even the most severe cases of mental illness, there is no consensus about how “advanced illness” might be conceptualized [18], and future research and discussion should address the extent to which the psychiatric profession is willing to discuss and accept such concepts.

In the advanced stages of physical illness, palliative care is often prioritized following discussion of goals of care by patients, significant others, and healthcare professionals in a process of shared decision-making [41]. In the case of SPMI, patients may themselves may be unable to press for a more palliative approach by virtue of their often impaired decision-making capacity (e.g. in cases of severe dementia) [42]. Advance directives are one possible remedy, but these are not currently widely implemented in psychiatry and often focus on other aspects of mental health care [43]. Consequently, health care professionals may shy away from “difficult discussions” and subsequent “difficult decisions” because they cannot rely on a clear mandate from the patient. Apart from suicide, death and dying seem rarely to be discussed in psychiatry, as indicated by the relative lack of relevant literature. Such attitudes may change if it can be shown that overly aggressive psychiatric care may be avoided by means of end-of-life discussions, as has previously been demonstrated for medical care [44].

The aim of PP is to improve the quality of life of patients with SPMI, who represent a particularly vulnerable population at risk of either therapeutic neglect or overly aggressive care. The new approach proposed here—an explicitly palliative approach in psychiatry—has the potential to improve quality of care, person-centredness, and autonomy for these patients. In contrast, under no circumstances should the term “palliative” be used to justify negligent or careless treatment of patients with SPMI. Furthermore, PP must not be seen to oppose the concept of recovery in psychiatry, which targets a similar group of patients. There are two models of recovery: clinical and personal. Clinical recovery “emerges from the concept of remission as an improvement in symptoms and functional deficits and implies the long-term goal of growing mental stability and psychosocial functioning with fewer or no relapses” [45]. Personal recovery focuses on fostering the process of personal development, growth, regaining control, and meaning in life despite SPMI [46], as for instance through peer support [47], supported employment and housing, or shared decision making [48]. PP might be expected to support the individual in reaching life recovery goals through self-determination and autonomy, dignity and acceptance. However, it is important that PP be understood as functioning in conjunction with other approaches oriented towards prevention, curation, rehabilitation, or recovery.

The lack of palliative care training opportunities for psychiatrists represents a considerable obstacle for the introduction of this approach to mainstream psychiatric thinking [21]. To date, there have been only pilot attempts during psychiatric residencies to increase exposure to end-of-life situations [49], and in postgraduate psychiatric curricula, any inclusion of palliative care lags behind other specialties [50, 51]. During their training, psychiatrists therefore have little opportunity to adopt a palliative care mindset that might inform their approach later in their careers. Mandatory rotations in outpatient chronic care, consultation and liaison psychiatric services, and geriatric psychiatry services may compensate in part for this lack of exposure, but specific efforts must be made to advance curriculum development in this regard.

## Conclusions and suggestions for future research

Despite psychiatrists’ efforts to prevent mental illness and to promote recovery, some patients will develop SPMI. These represent a particularly vulnerable population, at risk of either therapeutic neglect or overly aggressive care. As proposed here, a new variation on an old approach—the explicit application of palliative care principles to psychiatric illnesses—has the potential to improve quality of care, person-centredness, and autonomy for these patients. We hope that the working definition of PP suggested here may serve as a starting point for further development of a conceptual framework and clinical approach. Important milestones will include consensus around definitions of SPMI, palliative approaches, care decisions, and futility judgments, along with international acceptance of these concepts among psychiatrists and service users. Establishing an evidence-based staging model of mental illness may be an important prerequisite for these tasks.
